# Effect of emergent vegetation on riverbank erosion with sediment mining

**DOI:** 10.1038/s41598-024-61315-9

**Published:** 2024-05-16

**Authors:** Sukhjeet Arora, Bimlesh Kumar

**Affiliations:** Department of Civil Engineering, Indian Institute of Technology Guwahati (IITG), Guwahati, India

**Keywords:** Sand mining, Bank stability, Emergent vegetation, Shear stresses, TKE, Morphology, Hydrology, Limnology

## Abstract

The present work investigates the combined effects of the upstream sediment mining pit and vegetation on the riverbank using emergent rigid vegetation beyond the toe on the flow structure and morphological changes due to fluvial erosion. A steep gradient of streamwise velocity and other turbulence parameters such as Reynolds shear stress (RSS), transverse RSS, and turbulent kinetic energy (TKE) at the interface of the vegetated and unvegetated part of the test segment was observed. The cross-sectional analysis showed that vegetation increased the velocity of the unvegetated main channel, and the sandpit increased even the near-bed velocity with a similar trend in its longitudinal variation at the center line of the main channel. The abrupt variation in RSS and transverse RSS at the location of the berm induces instability and erodes the berm present at the toe of the riverbank. The combination of the vegetation and sandpit led to increased TKE of the flow at the near-bed and berm locations. The morphological analysis showed complete riverbank erosion in both cases of the unvegetated riverbank, i.e., without or with an upstream pit. The installed stems of rigid vegetation on the riverbank helped decrease the fluvial erosion of the riverbank, and its profile observed minimal changes over the length of the test segment. However, the main channel erosion was amplified due to the vegetation (in no-pit case) at the beginning of the test segment, which eroded the bed of the main channel by about 67% of the bed thickness. Also, in the vegetated riverbank cases, the upstream pit caused an increase in erosion by 7.66% at the center of the main channel. The study helps establish the hypothesis of negating the effects of sediment mining on bank erosion by using the rigid vegetation on the riverbank beyond its toe location, which performed well by maintaining the riverbank profile.

## Introduction

Riverbank erosion is a significant aspect of the complex river dynamics. The banks are the barriers that contain and regulate the river flow. A riverbank bears stresses from the interaction of the side slope with the flow, varying discharge over the year, freeze and thaw, different stages, diversion of flow, and anthropogenic activities such as the building of shore structures, sediment mining, waves from the passing boats, and reservoir projects upstream or downstream. These factors affect the sediment present on the bed and banks. In addition to the complexity of the sediment transport issue, riverbank instability raises the possibility of disastrous occurrences like floods^1^. Loss of valuable land area and channel migration would result from a failing riverbank^2–4^. The riverbed sediment is full of nutrients for the growth of crops. Also, the river sediment is helpful as a filler material in the concrete. The recent increase in urbanization has led to increased demand for sediments from the riverbed. Various illicit operators assume control over sediment mining operations, while governments regulate all other activities to some degree but have struggled to control illegal sediment mining^5,6^. Numerous organizations have said that 30–60 cm riverbed incision by mining is done annually, which may take even 25 years to recover when there is no further dredging activity^7,8^. Experts have stressed the importance of sediment dredging for socio-economic reasons, yet there is debate over the sustainability of sediment mining. Specifically, concerns have been raised about the negative ecological impacts resulting from indiscriminate dredging, including the potential loss of habitat for endangered crocodile species^9^. Research has shown that sediment mining causes an increase in the sediment transport rate and turbulence in the downstream flow^10,11^. These enhanced flow characteristics shall be adverse to riverbank erosion due to fluvial action. The mitigation action shifts towards stabilizing factors like spurs, installation of rocks of different sizes, river training structures, and increasing the riparian vegetation to minimize the detrimental effects of sediment dredging^12–15^.

Research has demonstrated that riparian vegetation shields the banks from erosion by changing the river's course and through root activity^16,17^. The vegetation at the riverbank slope interacts with the river flow. It diverts water from the bank towards the area not covered with vegetation in the main channel based on achieving the least resistance to the flow. This causes a slight rise in the water level in the vegetation zone, which happens when the flow attempts to overcome the plant's resistance to the flow in the flow region^18^. This increased water level changes the head in the vegetated and unvegetated parts of the cross-section^19^. This enables the transverse flow of the water away from the riverbank. This increases the average flow velocity of the main channel while slowing down the flow in the vegetated part of the cross-section. This slight increase in water depth near the bank may prove fatal if the flow water level approaches the flood danger levels^20–22^.

Numerous researchers have examined various vegetation types and plantation densities to determine how vegetation affects the river flow structure and morphology^23–28^. The class of vegetation is a substantial factor. It has been demonstrated that flexible vegetation exhibits more resistance to the flow than rigid vegetation^29^. Rigid vegetation has less effect on the flow than flexible vegetation. But, to protect the riverbank from erosion in a short-notice period for upcoming high flow rates suggested by hydrographs, installation/plantation of flexible vegetation may not be possible. In that case, any achievable protection will be beneficial to prevent the bank failure. The rigid vegetation should work similarly to flexible vegetation and divert the flow towards the main channel. However, it may accumulate debris and other solid waste flowing in the rivers^30^. This would result in further diversion of flow towards the main channel. The increased velocity in the main channel would enhance the riverbed erosion, facilitating sediment movement. The higher velocity will have more notable morphological consequences on the main channel^20,31^. Achieving the ideal plantation density is necessary to reduce the expense of artificially planting suitable vegetation. After working with a range of densities of different types of vegetation^16,32–34^, classified the density of vegetation as $a \times h < 0.1$ based on the height (*h*) and frontal area (*a*) of the vegetation in the flow. There might be zero, sparse, or dense vegetation in a river. According to earlier research, the main channel's velocity will increase as the vegetation's density increases^35,36^. Although the banks gain from installing vegetation, the main channel's flow is accelerated, severely eroding the riverbed. However, the riverbank has to be protected for safe passage of flood, for which vegetation needs to be installed as a natural and cost-effective method using the natural stems available or maybe introduce artificial stems with similar dimensions. The installation has to be done a little ahead of the anticipated floods when the riverbank is easily accessible due to the low stage of the river. The benefit of rigid vegetation over flexible vegetation would be that it can be installed even when the river stage is high, and the installation is the basic hammering of the rigid cylinders into the riverbank. During floods or periods of strong water flow, emergent rigid vegetation should shield the riverbank.

Anthropogenic activities, such as riverine structures, bridge piers, and sediment mining, affect the flow structure downstream and upstream of the source of disturbance. While these activities cannot be avoided, the location and extent of sediment mining should be regulated with a complete understanding of its effects on the hydrodynamics and morphology of the river. The altered flow structure caused by the sand mining pit has been studied in lab settings^10,32,37^. The impact of a sandpit on engineering structures such as tandem piers, oblong piers, and piers has been investigated by further researchers^11,38,39^. Studies on the impact of mining pits on riverbanks have been conducted in the past^2,40^. The benefits of vegetation on riverbanks have only been somewhat studied by researchers^36^. They studied the effects of vegetation density on the flow velocity in the asymmetric channel with a rigid bank slope. There is a significant gap in knowledge of the effects of two variables, such as the effects of sediment mining on the riverbank stability and the effects of erosion prevention techniques, such as planting rigid vegetation on the riverbank. The present study considers riverbank erosion for the riverbank stability and studies its effects in the presence of an upstream sandpit. It is worth noting that the study at hand is subject to certain limitations that should be acknowledged. One of these limitations is related to the available width of only 1 m, which may have affected the overall accuracy of the findings. Additionally, it is important to recognize that the assumption of a steady flow in the study is not always applicable to real-life scenarios, which may introduce some level of uncertainty into the results. Despite these limitations, the study provides valuable insights into the topic and lays a foundation for further research in this area.

Here, we study the flow's characteristics after it crosses the sandpit and how the pit's presence has affected the morphology of the test section. Further, we compare the hydrodynamic and morphological changes that occur on the riverbed and bank when emergent rigid vegetation has been installed along the bank from the top to the point slightly beyond the toe of the riverbank. The study aims to determine if rigid vegetation, which blocks a certain cross-sectional flow area along the riverbank, can help stabilize the riverbank when it has to counter the fluvial erosion due to the flow and the adverse effects of the upstream sediment mining pit. The flow structure's longitudinal and cross-sectional variations will help us understand the hydrodynamic effects of an upstream sediment pit on the vegetated riverbank and its impact on the unvegetated riverbank without (NPNV—No Pit and No Vegetation) and with an upstream pit (WPNV—With upstream pit and No Vegetation). The morphodynamic response of the vegetated riverbank section, a channel segment with a side slope, without (NPWRV—No Pit and With Rigid Vegetation) or with an upstream sandpit (WPWRV—With upstream Pit and With Rigid Vegetation), will help understand the effects of sediment dredging and emergent rigid vegetation as a mitigating factor to check riverbank erosion.

## Materials and methods

**Figure 1:**
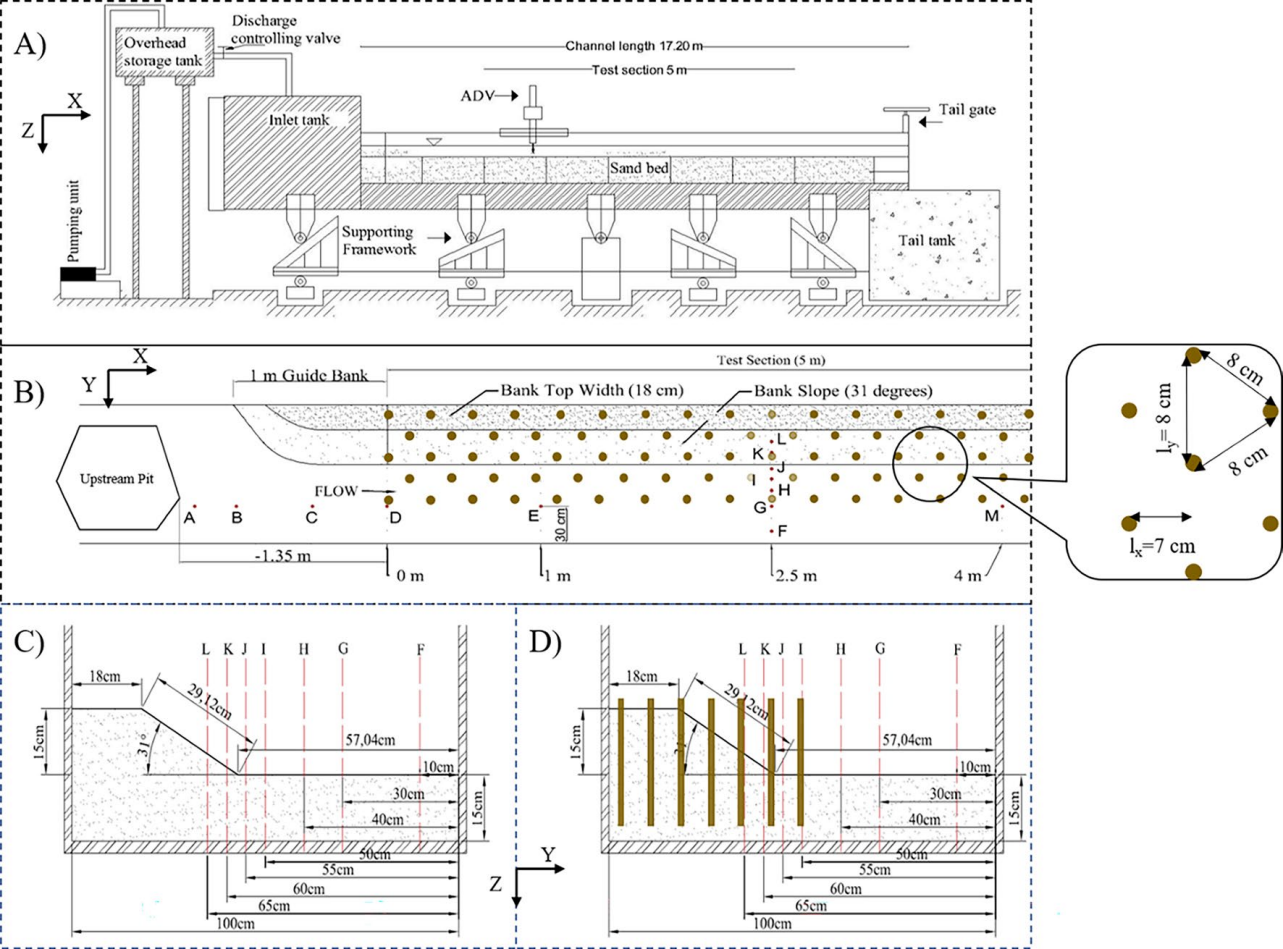
Laboratory setup presenting (**A**) side view; (**B**) top view with upstream pit and rigid vegetation in staggered pattern; (**C**) cross-section at 1 m after entrance into the test section without vegetation; (**D**) cross-section with rigid vegetation.

For this investigation, a 1 m deep, 1 m wide, and 17.2 m long flume was used to conduct each experiment for 24 h (Fig. 1A). Two 5.6 kW centrifugal pumps, which work to enable the flow recirculation system, were installed in the setup. These pumps intake water from a large sump tank with a 60 m^3^ capacity into an overhead supply tank (2.3 m × 1 m × 1 m). Water from the supply tanks is then supplied into the upstream head tank of the rectangular flume via a large pipe (0.23 m in diameter). In order to guarantee a seamless transition upon entering the experimental flume, the flow was directed through baffle barriers. The supply pipe's single butterfly valve controlled the channel's flow rate. Progressive and gradual pumping was used to introduce water into the channel for each test, and this valve was used to increase discharge until the desired flow rate was reached. After passing through the downstream rectangular notch, which measures the flow rate, the flow left the experimental flume and headed toward the main underground sump for recirculation. The initial 7.0 m flume was kept to attain the fully developed turbulent flow conditions before reaching the test segment^11,41^. A 0.15 m deep evenly graded sand bed with a geometric standard deviation of 0.93 and a median size ${d_{50}} = 0.3\;{\text{mm}}$ was spread out over the whole length of the flume bed. In order to confirm the same degree of compaction in each experiment, the sand was added without any drops, and the channel was filled with water and drained. The sandbed was found to be compacted due to the experimental run. This procedure of preparation was adopted to reset the bed. Then, using a wooden mold driven down the channel, the placed sand was only trimmed to the required 31° riverbank slope (angle of repose) and cross-sectional dimensions. The test segment measured 5.0 m in length, along with guide banks for smooth flow entry and exit (Fig. 1B). The riverbank guide banks start from zero width and gradually increase to match the width of the top of the riverbank. The riverbank's cross-section measured 0.18 m in top width, 0.15 m in unsupported side slope height, and the width of the main channel was 0.58 m, as shown in Fig. 1C. The flow depth (*h*) was maintained at 0.14 m. The Froude's number was within the sub-critical range (Table 1). Throughout the 24-h experimental run, there was no external sediment delivery to the test system, and the water was clear of any suspended sediment load. There are two side slopes in a river, but only one side slope was prepared here since both symmetrical halves were assumed to have the same flow structure^42,43^.

**Table 1:** Conditions pertaining to the experimental flow.

S. no.	Pit/vegetation	Case name	Discharge (m^3^/s)	Froude’s number
1	No pit, no vegetation	Incipient condition	0.023	0.239
2	No pit, no vegetation	NPNV	0.030	0.307
3	With pit, no vegetation	WPNV	0.030	0.291
4	No pit, with rigid vegetation	NPWRV	0.030	0.366
5	With pit, with rigid vegetation	WPWRV	0.030	0.349

### Instrumentation and data recordings

**Figure 2:**
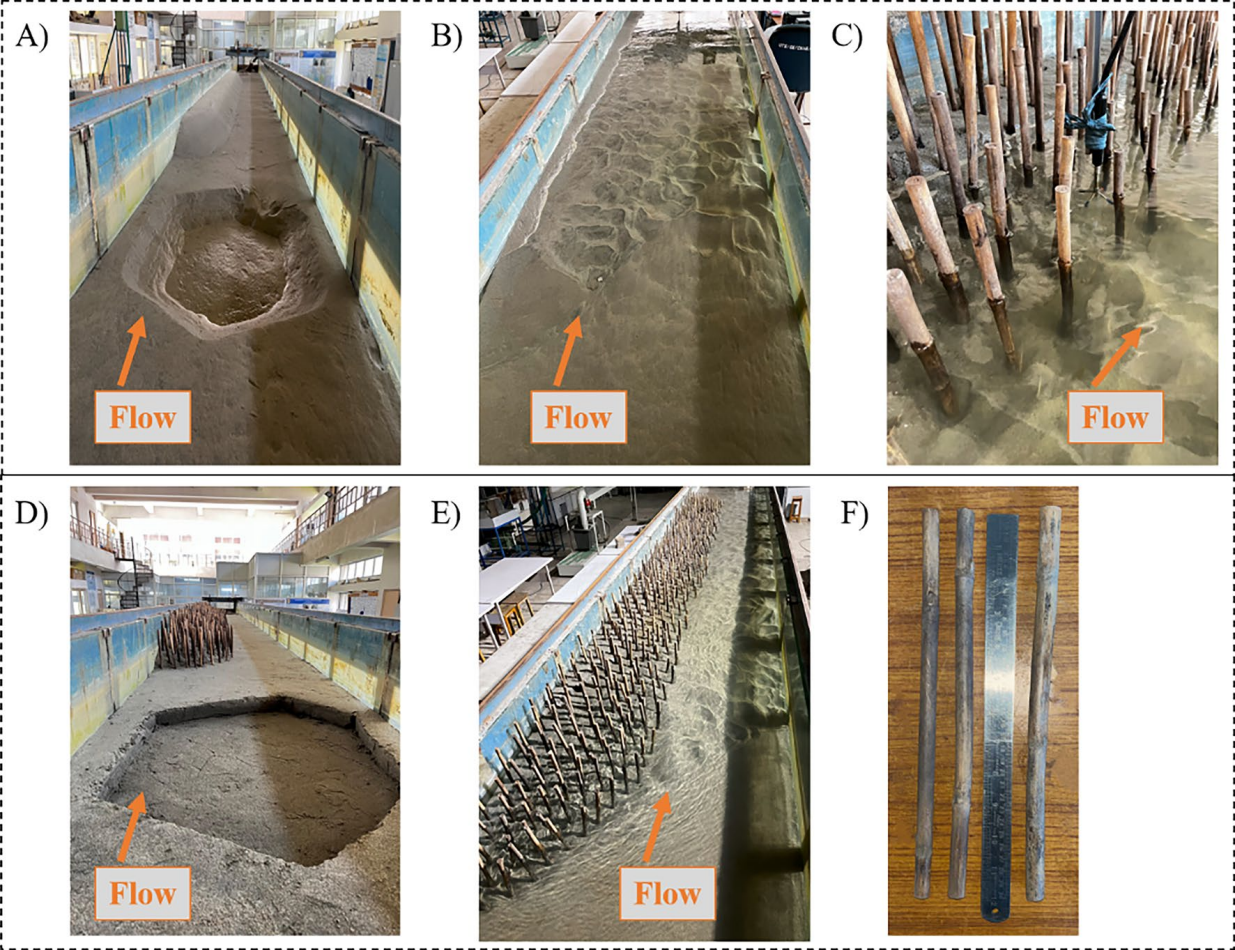
Pictures of experimental setup (**A**) case WPNV, which will be NPNV without sediment pit at given location; (**B**) during the experimental run for no-vegetation cases showing eroded riverbank; (**C**) usage of ADV probe in cases of vegetated riverbank; (**D**) case WPWRV which will be NPWRV without sediment pit at given location; (**E**) during the experimental run of vegetated riverbank showing stable riverbank profile (**F**) stems of rigid vegetation kept next to 30 cm steel scale.

In the sediment channel, four sets of experiments were conducted: one set had no upstream mining pit (NPNV), one set had an irregularly shaped upstream mining pit that was 0.075 m deep (WPNV) (Fig. 2A,B), and the other two sets used the same case of no-pit (NPWRV) and an upstream pit (WPWRV) of same size and shape along with the rigid natural vegetation cylinders of perennial reed (Phragmites karka) at the riverbank in the test section (Fig. 2C–E). The test section achieved a continuous flow discharge of 0.03 m^3^/s without digging a pit. The flow interacted with the riverbank test section during the experimental runs, eroding the river bed and bank sediments. The quasi-equilibrium was reached within two hours of the start of the experiments. The tests were carried out until the transport rate stopped and no sediment movement was observed. Each experiment was exposed to the same fluvial activity for 24 h. An irregularly shaped upstream pit measuring 0.075 m in depth, 0.7 m in width, and 0.85 m in length was excavated, which ended 1.35 m upstream of the test section for the dredging scenario (Fig. 1B). The asymmetrical structure of the mining pit was chosen to emulate actual situations when pits of varying shapes are created during sand mining in rivers. The pit's dimensions and form were maintained throughout this study to allow for an equitable comparison of the turbulence characteristics across all cases. The with-pit case tests were carried out at the same flow discharge rate of 0.03 m^3^/s as the no-pit test. As seen in Fig. 1D, natural cylindrical stems of diameter 0.01–0.015 m were driven into the riverbank to investigate the combination of the adverse effects of the sand mining pit and erosion prevention effects of vegetation on the riverbank. The hydraulic parameters for each experiment are listed in Table 1. The natural cylinders were installed vertically from the top of the bank to the center of the test section ($Y \leqslant 0.5\;{\text{m}}$). The cylinders were 0.3 m long rigid stems of perennial reed (Phragmites karka) and were placed with 0.14 m penetration into the sand bed (Fig. 2F). Based on the literature, all the stems were placed at an equal distance of 0.08 m center-to-center apart from each other in a staggered pattern to achieve compact vegetation density^23,44–46^. As shown in Fig. 1B, the average spacing reached by measuring the plant density at various cross-sections was ${l_Y} = 0.08\;{\text{m}}$, and the vegetation streamwise spacing throughout the test segment was ${l_X} = 0.0692 \approx 0.07\;{\text{m}}$.

**Figure 3:**
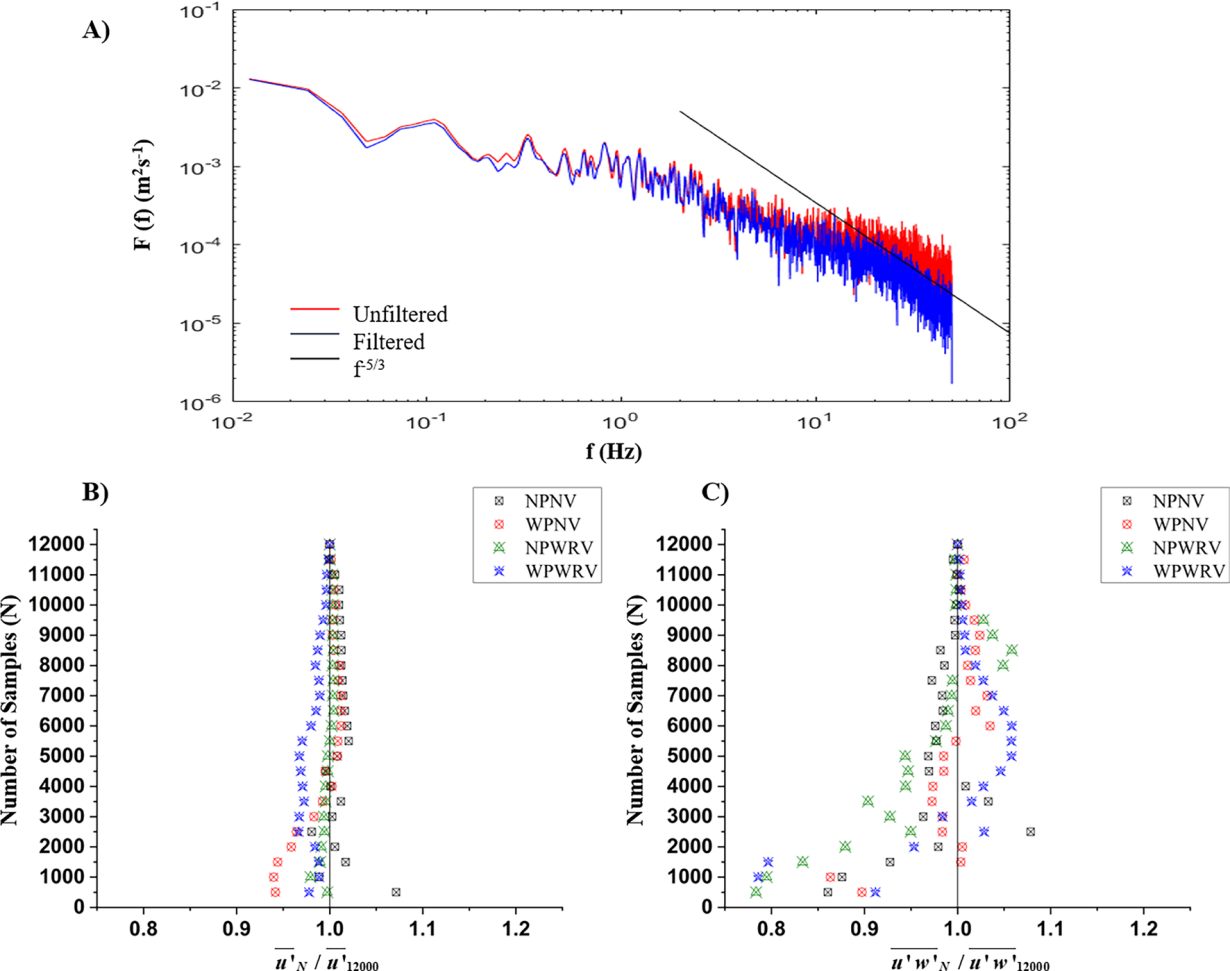
(**A**) Velocity power spectral function for the streamwise velocity showing unfiltered, filtered and the − 5/3 power law line given by the Kolmogorov's − 5/3 power law; Convergence analysis of (**B**) streamwise velocity (**C**) RSS.

Turbulence velocity data were collected at seven sites in a cross-section 2.5 m downstream of the flow entrance into the test segment (Fig. 2C). The velocity time series at these site locations (F–L) for cross-sectional variations and at points (A–E, G, M) for longitudinal variations were recorded. The three-dimensional flow velocity was taken using the Nortek Acoustic Doppler velocimeter (ADV), as shown in Fig. 1B. It is a four-beam, fixed-stem, down-looking probe with a maximum sampling rate of 200 Hz, called the Vectrino model. For every observation point along the depth of an observation site, a minimum of 120 s of velocity time series were captured at 100 Hz, which gives us 12,000 sample readings. The ADV observes the cylindrical-shaped sampling volume of 1 cm^3^ at the vertically downward distance of 5 cm. The convergence analysis was done to ascertain the sufficiency of the samples for attaining the mean of turbulent parameters. It was conducted on streamwise velocity and RSS by taking the average of the first *N* number of samples and studying the non-dimensionalized average of streamwise velocity and RSS for their overall average with 12,000 samples (Fig. 3B,C). The convergence analysis indicated that streamwise velocity converged at 7000 samples with an error percentage of less than 2%, and RSS converged at 9000 samples with an error percentage of less than 4%. It indicates that 12,000 sample readings at an observation point were sufficient to keep the error within the acceptable range^47,48^. The velocities u, v, and w obtained from ADV were correlated to the flow direction, transverse direction, depth-wise, or along the X, Y, and Z axes, respectively. The velocity measurements contained noise spikes. The acceleration threshold technique was used to despike to get a clean velocity time series^20^. The spikes are identified with by observing their acceleration from the immediate neighbour. The instantaneous velocity reading that shows acceleration or retardation of more than the acceleration due to gravity has been considered as noise. The noise value is then replaced by linear average of its immediate neighbours. Table 2 enumerates the uncertainties associated with velocity measurements obtained in the experimental studies. At location G, which is in the inner layer of the main channel flow, the velocity power spectra (*F*(*f*)) of the streamwise velocities and frequency *f* are shown in Fig. 3A. According to Kolmogorov's theory, the velocity power spectra in the inertial subrange of isotropic turbulence obey the “− 5/3” rule. The unfiltered and filtered streamwise velocity spectra are given in Fig. 3A. The filtered (given in blue) velocity spectra align well with the given slope line − 5/3. The acceleration threshold value was found by trial and error to be between 1 and 1.5^49,50^.

**Table 2:** Analysis of uncertainty in velocity measurements.

(m/s)	u	v	w	(u′u′)^0.5^	(v′v′)^0.5^	(w′w′)^0.5^
Standard deviation	$4.09 \times {10^{ - 3}}$	$8.88 \times {10^{ - 4}}$	$3.46 \times {10^{ - 4}}$	$1.57 \times {10^{ - 3}}$	$1.65 \times {10^{ - 3}}$	$2.39 \times {10^{ - 4}}$
Uncertainty %	0.24	0.07	0.08	0.06	0.047	0.028

## Results and discussion

The flow structure was studied using streamwise velocity variations, Reynolds shear stress (RSS), transverse RSS, and Turbulent Kinetic Energy (TKE). The instantaneous velocity data for the entire flow depth was obtained longitudinally at $Y = 0.7\;{\text{m}}$ and at the cross-section 2.5 m after entering the test section at points ‘A–M’ for each test case to comprehend the flow structure across the whole riverbank test segment. The result profiles exclude a 0.05 m wide zone next to the glass wall to exclude the side-wall impacts. Additionally, morphological changes in the riverbank's cross-section, 2.5 m after the start of the test segment and along the length $Y = 0.7\;{\text{m}}$ caused by flow, were examined for all four cases. This section presents the test case findings and discusses their effects on the morphological changes of the test segment.

### Mean velocity

**Figure 4:**
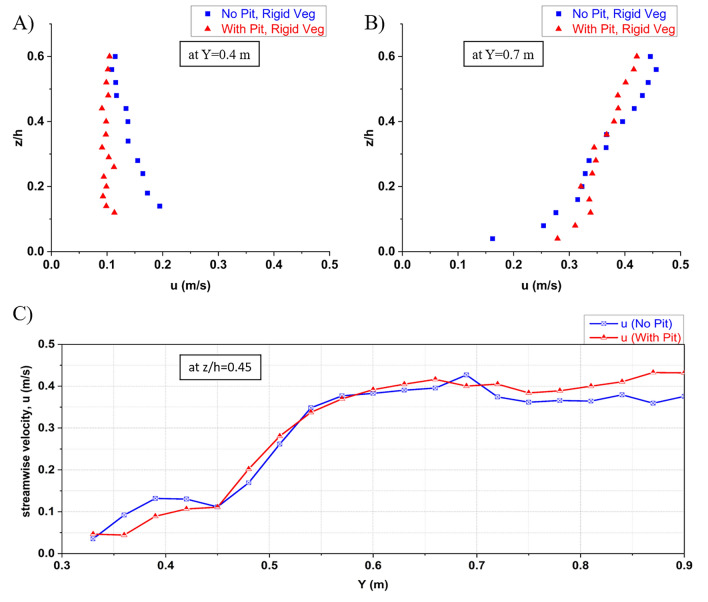
Streamwise velocity variation of the cases of NPWRV and WPWRV (**A**) at the location J, Y = 0.4 m, within the vegetated segment; (**B**) at the location G, Y = 0.7 m, at the center of the main channel; (**C**) across the cross-section, at a depth of z/h = 0.45, at a distance of 3 cm apart from each other, representing the accuracy of variations presented in contours of Fig. 5

According to Reynolds decomposition, the mean ($$ \overline{\text{u}}$$, $$ \overline{\text{v}}$$, and $$ \overline{\text{w}}$$) and its fluctuating velocity components (u′, v′, and w′) make up the whole of the turbulent velocities streamwise (u), transverse (v), and depthwise (w). Figure 4A,B shows the variation of streamwise velocity along the vegetated riverbank's depth at $$ Y = 0.45\;{\text{m}}$$ and at $$ Y = 0.7\;{\text{m}}$$, respectively, for no-pit (NPWRV) and upstream-pit (WPWRV) cases. The streamwise velocity seems to be constant for the entire depth in the vegetated zone of the riverbank cross-section ($$ Y = 0.45\;{\text{m}} < 0.5\;{\text{m}}$$). The logarithmic law is followed in the unvegetated part of the test section ($$ Y = 0.7\;{\text{m}} > 0.5\;{\text{m}}$$). An increase in near-bed streamwise velocity in the unvegetated main channel was observed due to the sandpit, which, in contrast, decreased in the vegetated riverbank zone. This indicates further slowing down of the near-bank flow, which will help mitigate the fluvial erosion of the bank. However, the increased difference in velocity at the interface in the near-bed zone ($$ z/h < 0.2$$;$$ Y = 0.5\;{\text{m}}$$) will eventually result in more shear stresses and vortices formation^51^. This increased turbulence at the interface will erode the berm at the riverbank toe. Figure 4C presents the variation of the streamwise velocity along the cross-section at the intervals of $$ \Delta Y = 0.03\;{\text{m}} $$ at a fixed depth $$ z/h = 0.25$$. It helps understand the pattern of change in velocity in the vegetated zone ($$ 0\;{\text{m}} < Y \leqslant 0.5\;{\text{m}}$$), unvegetated zone ($$ 0.5\;{\text{m}} < Y < 1\;{\text{m}}$$), and at their interface for both the cases of no-pit and with-pit. It can be inferred that the streamwise velocity in the vegetated zone further decreased for the with-pit case. At the same time, a slight increase is observed in the main channel, which would help mitigate the fluvial erosion of the riverbank, but at the cost of increased erosion in the main channel.

**Figure 5:**
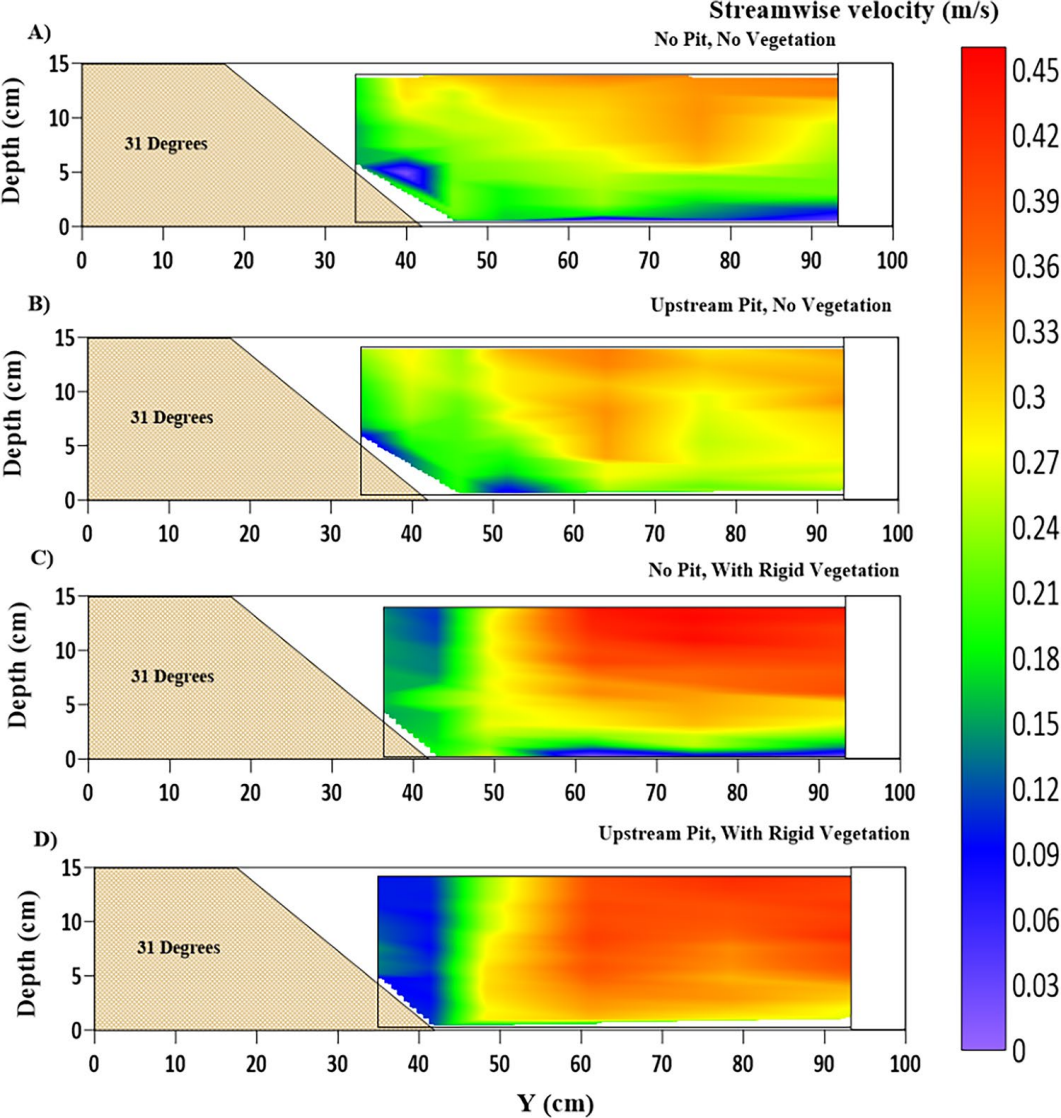
Contour of mean streamwise velocity ($\overline{\text{u}}$) (m/s) fluctuations at the cross-section located 2.5 m after the beginning of the test section, where (**A**) NPNV; (**B**) WPNV; (**C**) NPWRV; (**D**) WPWRV.

Figure 5 depicts the contours of the streamwise velocity (m/s) at the cross-section 2.5 m after the entrance of the test segment for all four cases of this study, along with a common color scale for its magnitude. An apparent increase in near-bed streamwise velocity for both no-vegetation (Fig. 5A,B) and with-rigid-vegetation (Fig. 5C,D) due to the sand pit can be observed. Also, the sand pit has caused a reduction in the near-bank streamwise velocity. These changes cause training of flow away from the bank and cause movement of the riverbeds and berms' eroded material downstream. For the ongoing current flow conditions, it will assist in stopping riverbank erosion, but as prior studies have shown, it will result in a higher unsupported height of the riverbank for the upcoming seasons^2,41^. Higher mean streamwise velocity gradients result from sand dredging in the near-bank zone (location K). It suggests that the boundary layer created in the near-bank flow would experience more viscous shear due to the pit^11^.

**Figure 6:**
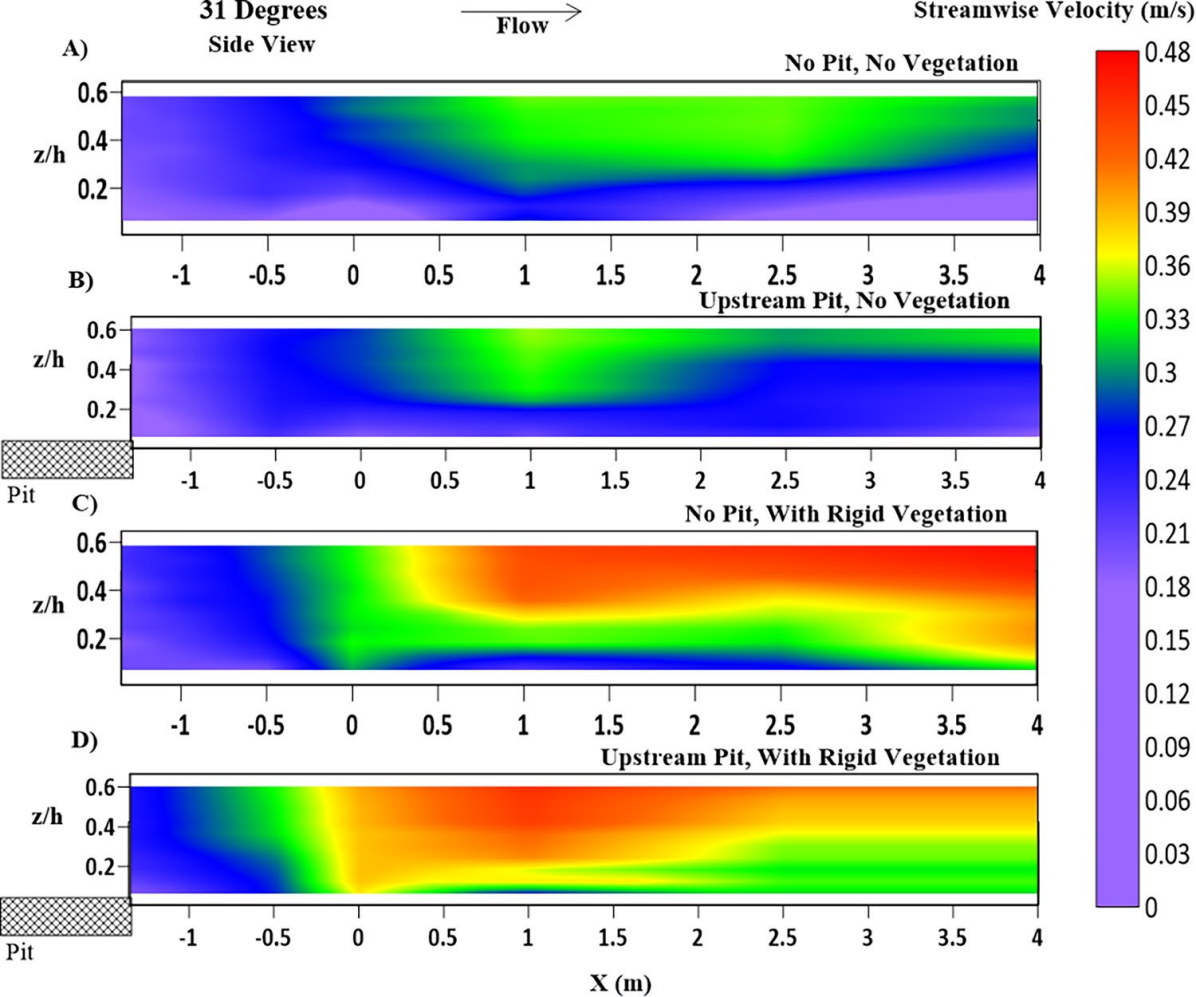
Contour of mean streamwise velocity ($\overline{\text{u}}$) (m/s) variations, at Y = 0.7 m, at the center line of the main channel representing (**A**) NPNV; (**B**) WPNV; (**C**) NPWRV; (**D**) WPWRV.

Figure 6 shows the longitudinal variation of the streamwise velocities versus the normalized flow depth ($$z/h$$) in the riverbank, located in the main-channel section at $$ Y = 0.7\;{\text{m}}$$, with the inner layer reaching $$ z/h = 0.1 - 0.25$$. In with-pit settings, the sandpit in the main channel caused bed sediment erosion and a slight rise in water depth. Consequently, mean streamwise velocities in the outer layer ($$ z/h > 0.2$$) decrease in the case of a channel pit. In the region ($$ z/h < 0.2$$), the velocity increases downstream of the sandpit, as shown in Fig. 6B,D. The increase in the near-bed streamwise velocity due to the sandpit is evident in Fig. 6A,B. However, this increase in streamwise velocity is more in the cases of vegetated riverbanks as the main channel has to accommodate the flow diverted away from the riverbank by the vegetation. As seen in profile Fig. 6C, the vegetation along the riverbank causes an increase in the streamwise velocity as the flow constricts from the rectangular cross-section before the start of the test segment ($$ - 1.35\;{\text{m}} < X < 0\;{\text{m}}$$). In the case of WPWRV, as seen in profile 6D, riverbank vegetation causes the flow to accelerate in the main channel, while the sandpit causes acceleration in the near-bed region. The WPWRV case in Fig. 6D ($$ Y = 0.7\;{\text{m}}$$) has a higher streamwise velocity beyond the entrance of the test section ($$ X > 0\;{\text{m}}$$) due to the presence of an upstream sediment pit, which causes a slight increase of head when the flow crosses the pit as observed by the previous researchers^10,11,38^.

### Reynold's shear stress

**Figure 7:**
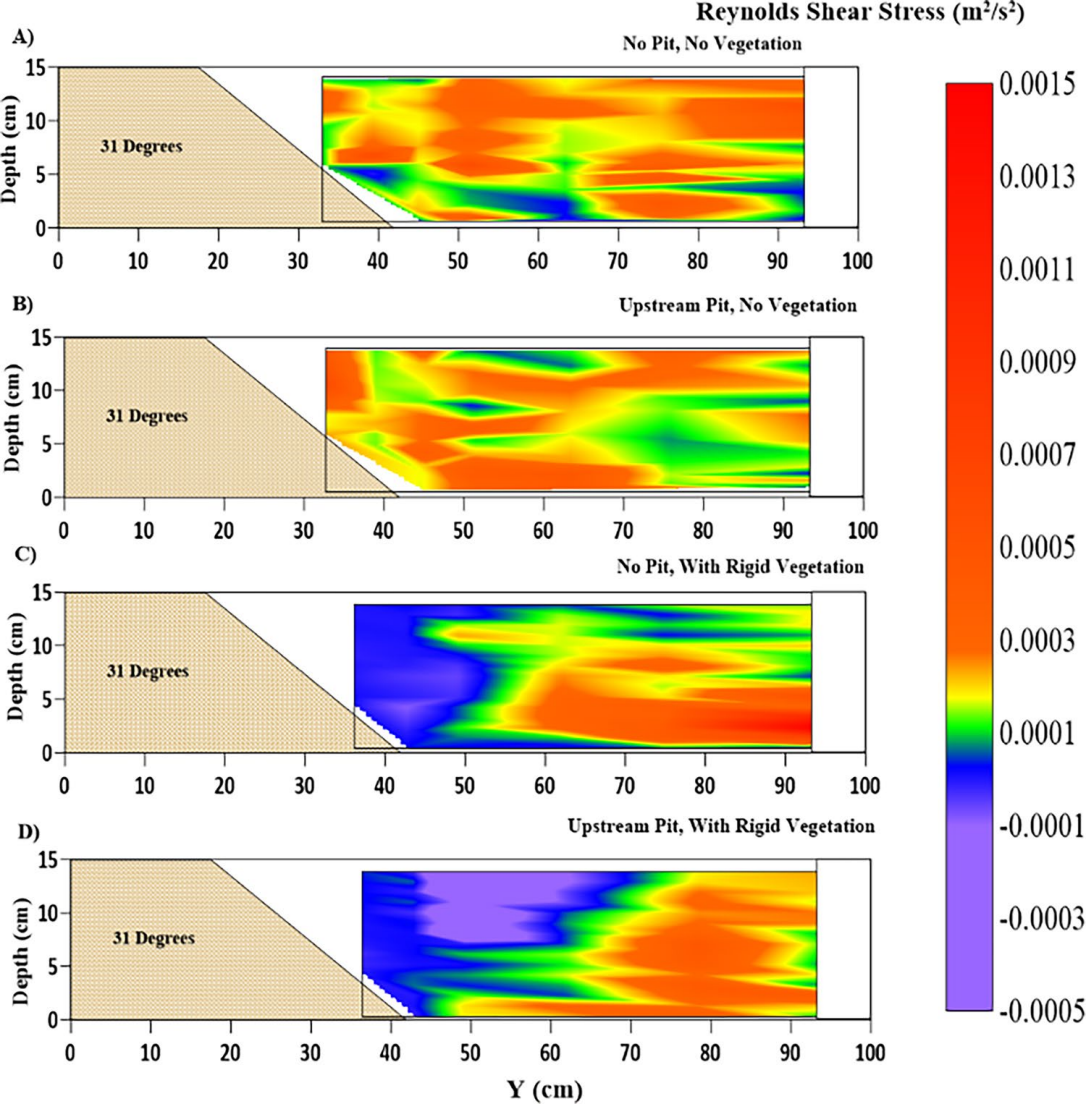
Contour of Reynolds shear stress ${\tau_{Z - X}}$ (m^2^/s^2^) variations at the location of 2.5 m after the beginning of the test section representing (**A**) NPNV; (**B**) WPNV; (**C**) NPWRV; (**D**) WPWRV.

**Figure 8:**
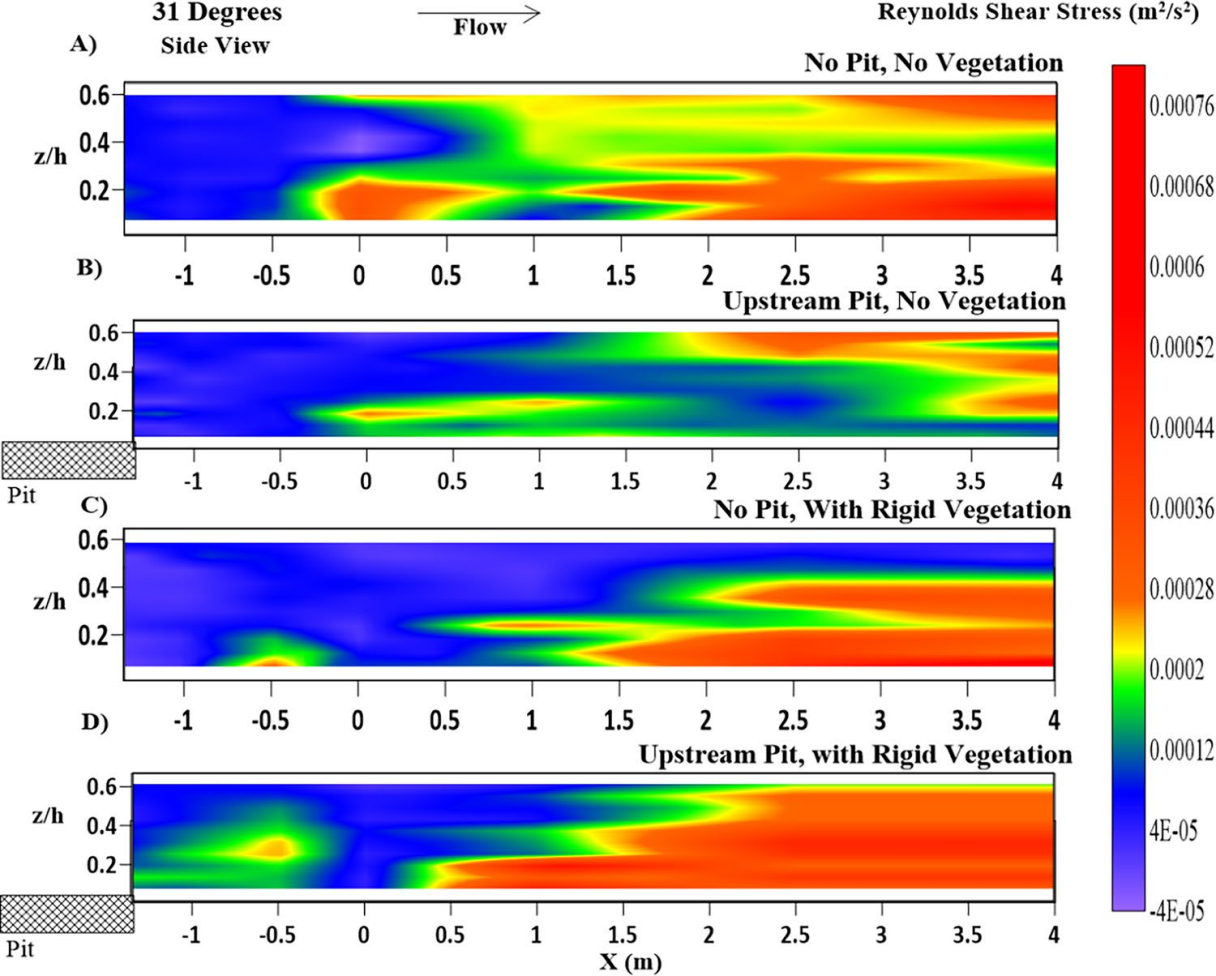
Contour of Reynolds shear stress ${\tau_{Z - X}}$ (m^2^/s^2^) longitudinal variations, at Y = 0.7 m, at the center line of the main channel representing (**A**) NPNV; (**B**) WPNV; (**C**) NPWRV; (**D**) WPWRV.

In every test condition, the flow in the riverbank segment is highly turbulent and rough $$ {{\text{R}}_e} > 10{,}000$$. Eulerian measurements of the flow's turbulent velocities are used in this study. It refers to the instantaneous velocity observations taken at the given sections. Flow on the bank slope controls silt movement and fluvial erosion on the slope after the sliding failure. Mass collapse and fluvial erosion are the two leading causes of riverbank failures. The turbulence shear forces acting on the riverbank sediments are essential as they lay on the X–Y plane because these stresses directly influence the movement and deposition of sediments. These two turbulent shear components, operating in streamwise and transverse directions on the X–Y plane of sediments, are $$ {\tau_{Z - X}}$$ and $$ {\tau_{Z - Y}}$$, respectively, where $$ {\tau_{Z - X}} = - \overline {u^{\prime}w^{\prime}} $$ and $$ {\tau_{Z - Y}} = - \overline {u^{\prime}v^{\prime}} $$ (m^2^/s^2^). Here, u′ and w′ are obtained from the experimental readings using ADV, as mentioned in Materials and Methods above. These shear forces give the sediment a push in the streamwise and transverse directions, respectively. This helps the sediment particles to overcome the forces of resistance. If the shear forces are sufficient enough to reach the incipient condition, then the sediment particles start moving. Reynolds stresses contours on the plane X–Y in the streamwise direction in the Cartesian coordinate system are depicted in Figs. 7 and 8, showing cross-sectional and longitudinal variations, respectively. The NPNV case in Fig. 7A suggests a zone of high shear stresses concentrated in the outer layer zone ($$ z/h > 0.2$$). The near bank RSS causes riverbank erosion, which has flattened the cross-sectional profile, as shown in Fig. 12. The WPNV case in Fig. 7B shows the concentration of the high-RSS region in the near bed zone ($$ z/h < 0.2$$), which leads to increased sediment migration. The streamwise RSS at the berm location ($$ 0.4\;{\text{m}} < Y < 0.6\;{\text{m}}$$) has increased significantly due to the presence of the upstream sandpit, which leads to unstable slopes and increases the unsupported height of the riverbank. The high fluctuations in streamwise RSS in Fig. 7C,D suggest intermixing of the flow moving downwards (positive values) and upwards (negative values), respectively. These fluctuations have become stronger in the WPWRV case than the NPWRV case, leading to higher turbulence in the flow and, eventually, increased riverbed erosion volume, as shown in Fig. 13C,D.

Longitudinal Profiles of RSS at $Y = 0.7\;{\text{m}}$, in Fig. 8A,B reveal that the pit causes a decrease in streamwise RSS at the center line of the cross-section while it has increased significantly in the near-bank region, as seen in Fig. 7A,B. The presence of vegetation in NPWRV and WPWRV cases was observed to have higher streamwise RSS in the main channel as compared to the NPNV and WPNV cases due to the increased flow velocity and its fluctuations in the unvegetated main channel of the test segment. The upstream sediment pit has shown an evident increase in the streamwise RSS, as seen in Fig. 8D, compared to Fig. 8C, which assists the sediment transport. The lateral or transverse shear stresses become an important factor in studying flow characteristics in a vegetated test segment as flow moves away from the riverbank slope in the lateral direction.

### Transverse RSS

**Figure 9:**
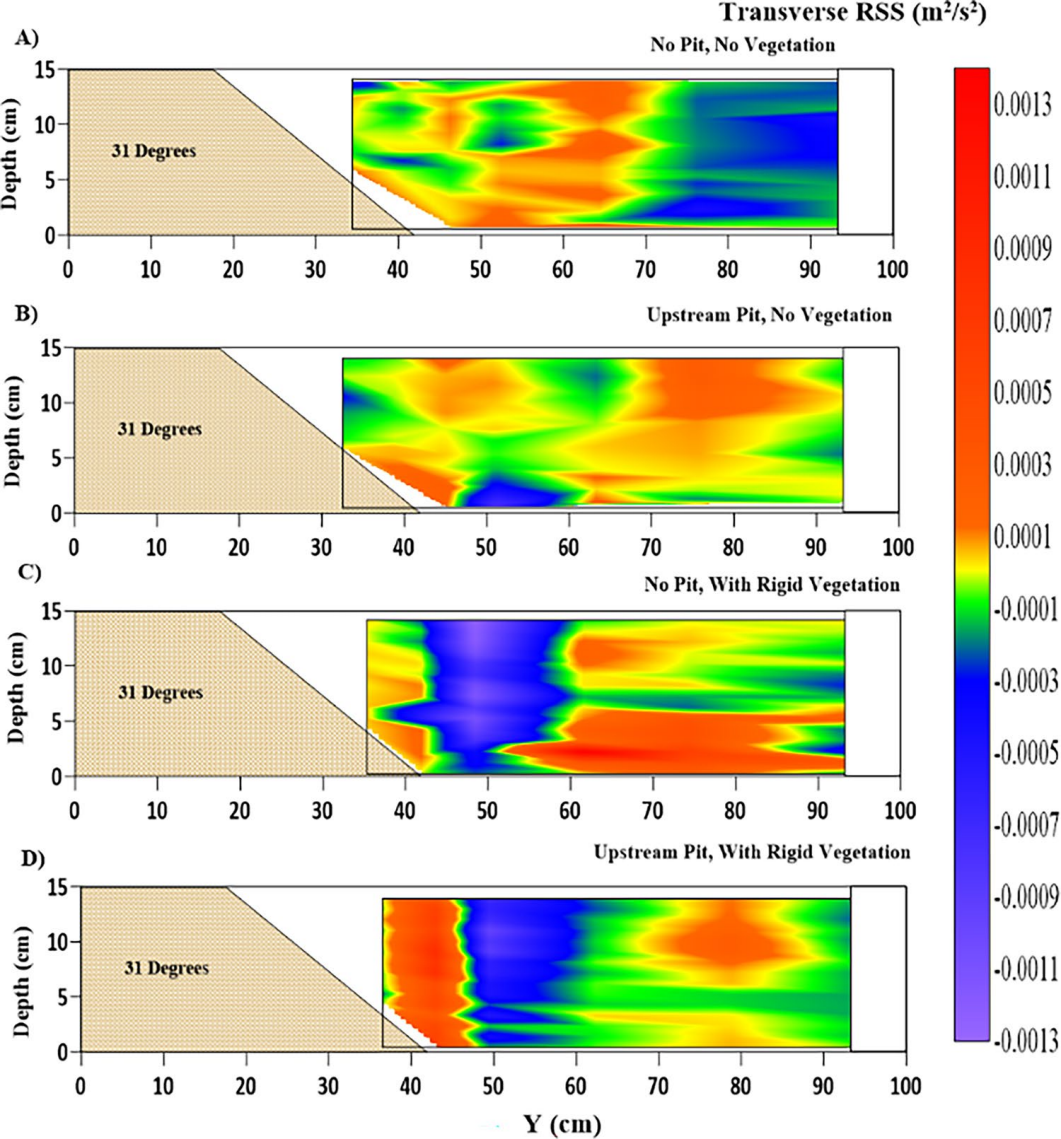
Contour of transverse shear stress ${\tau_{Z - Y}}$ (m^2^/s^2^) variations at the location of 2.5 m after the beginning of the test section representing (**A**) NPNV; (**B**) WPNV; (**C**) NPWRV; (**D**) WPWRV.

The contours of transverse RSS (${\tau_{Z - Y}} = - \overline {u^{\prime}v^{\prime}}$) across the cross-section are presented in Fig. 9. The transverse RSS, with fluctuating components of streamwise and transverse velocities, highlights the lateral stresses acting in the cross-section, gives vital details of the flow structure, and helps understand the morphological behavior of the riverbank and riverbed. It should be noted that the transverse RSS and streamwise RSS are similar, which states the importance of studying the transverse RSS in this setup. The highly fluctuating transverse RSS in Fig. 9 represents the higher inter-mixing of the flow in the cross-section. This destabilizes the cross-sectional profile of the riverbank and riverbed by causing increased sediment instability and its transport in the lateral direction. Figure 9A, positive transverse RSS in $0.35\;{\text{m}} < Y < 0.7\;{\text{m}}$ suggests the lateral stresses in the flow in the direction away from the riverbank towards the main channel. The upstream sediment pit in Fig. 9B has caused a decrease in the transverse RSS as it increases the streamwise RSS, as shown in Fig. 7B. The rigid vegetation on the riverbank at $Y \leqslant 0.5\;{\text{m}}$ causes the formation of vortices by creating a velocity gradient in the vegetated and unvegetated segment, as shown in Fig. 4C. The vegetation has led to very high transverse RSS in the zone $Y > 0.6\;{\text{m}}$, which has adverse effects on the stability of the main channel, as seen in Fig. 12. Although the sediment pit in the WPWRV case, as shown in Fig. 12D, has alleviated the transverse stress in the main channel, it has led to a sharp increase of transverse RSS near the riverbank. It may have occurred due to the increased streamwise fluctuations in the flow due to the pit's presence. Therefore, high streamwise and transverse RSS shifts sediment loads and contributes to the degradation of these riverbank slopes' channel banks and main-channel erosion.

### Turbulent kinetic energy (TKE)

**Figure 10:**
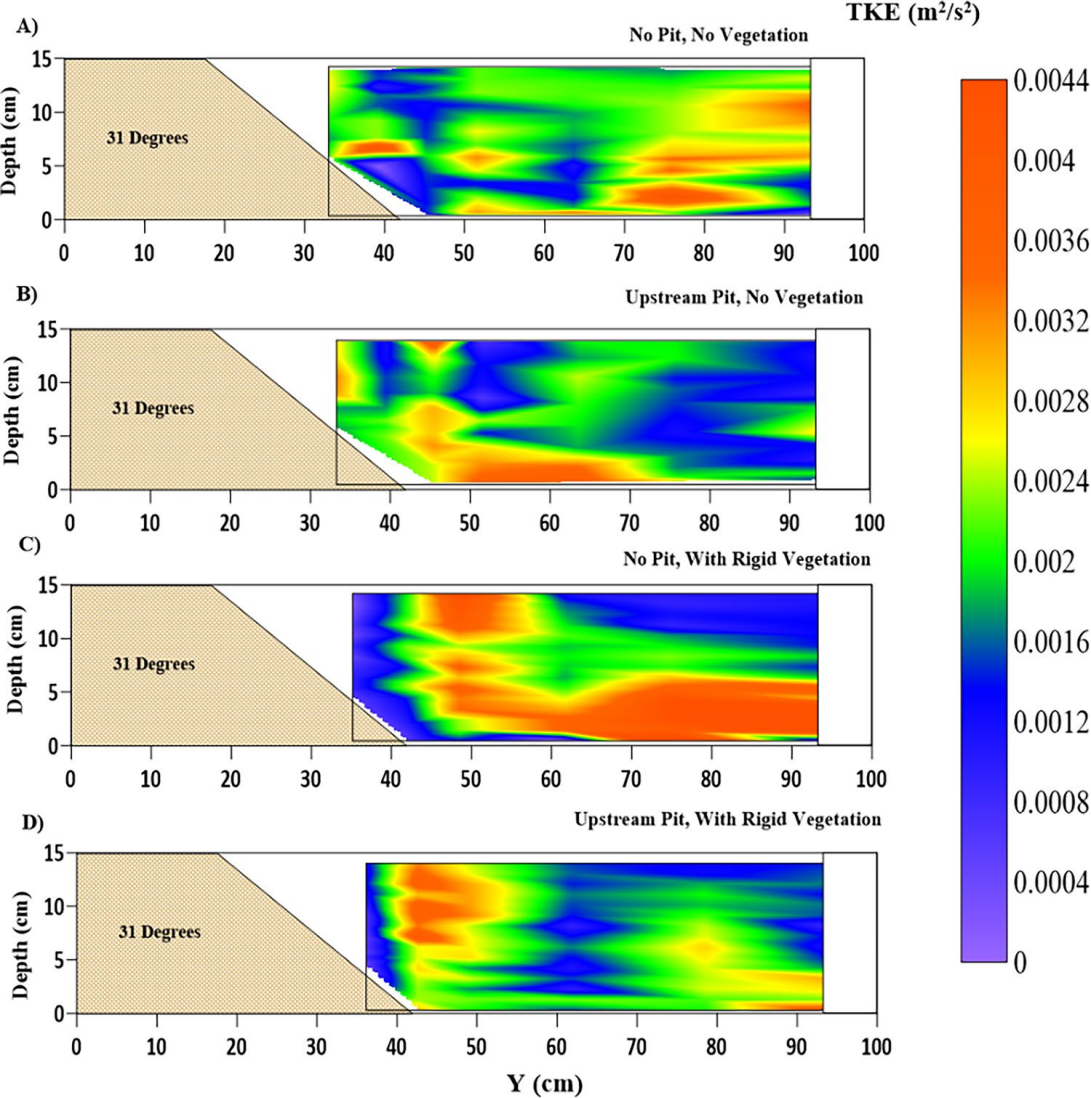
Contour of turbulent kinetic energy (TKE) k (m^2^/s^2^) variations at the location of 2.5 m after the beginning of the test section representing (**A**) NPNV; (**B**) WPNV; (**C**) NPWRV; (**D**) WPWRV.

The TKE, $k = 0.5 \times (\overline {u^{\prime}u^{\prime}} + \overline {v^{\prime}v^{\prime}} + \overline {w^{\prime}w^{\prime}} )$, at the cross-section for all four cases, has been presented in Fig. 10. The TKE represents the sum of flow intensities in three directions of flow. Increased TKE values shall indicate an increase in the overall turbulence energy of the flow. This indicates an increase in the sediment erosion capacity of the flow. The turbulent kinetic energy is distributed unevenly across the cross-section. However, it is consistent throughout the depth at different cross-section points and with higher turbulence in the main channel for the NPNV case (Fig. 10A). The upstream pit has caused an increase in the turbulence at the location of the berm for the WPNV case (Fig. 10B). It has caused a shift of the high turbulence region towards the near-bed region. It would cause an increase in the erosion of the berm and bed. It would lead to the increased unsupported height of the bank, which supports the results of previous studies^41^. Figure 10C suggests a sharp increase in turbulence levels at the location of the vegetation interface ($Y = 0.5\;{\text{m}}$) for the case NPWRV. The higher turbulent kinetic energy near the bed may have occurred due to the increased contribution of the lateral components of TKE. Figure 10D shows higher turbulence at the interface and the near-bed region of the main channel for $Y > 0.7\;{\text{m}}$. The decreased depthwise turbulence at $Y = 0.6\;{\text{m}}$ may have happened due to the simultaneous occurrence of the vegetated riverbank and the upstream sandpit and resulted in less erosion as seen in Fig. 12C at $Y = 0.6\;{\text{m}}$.

**Figure 11:**
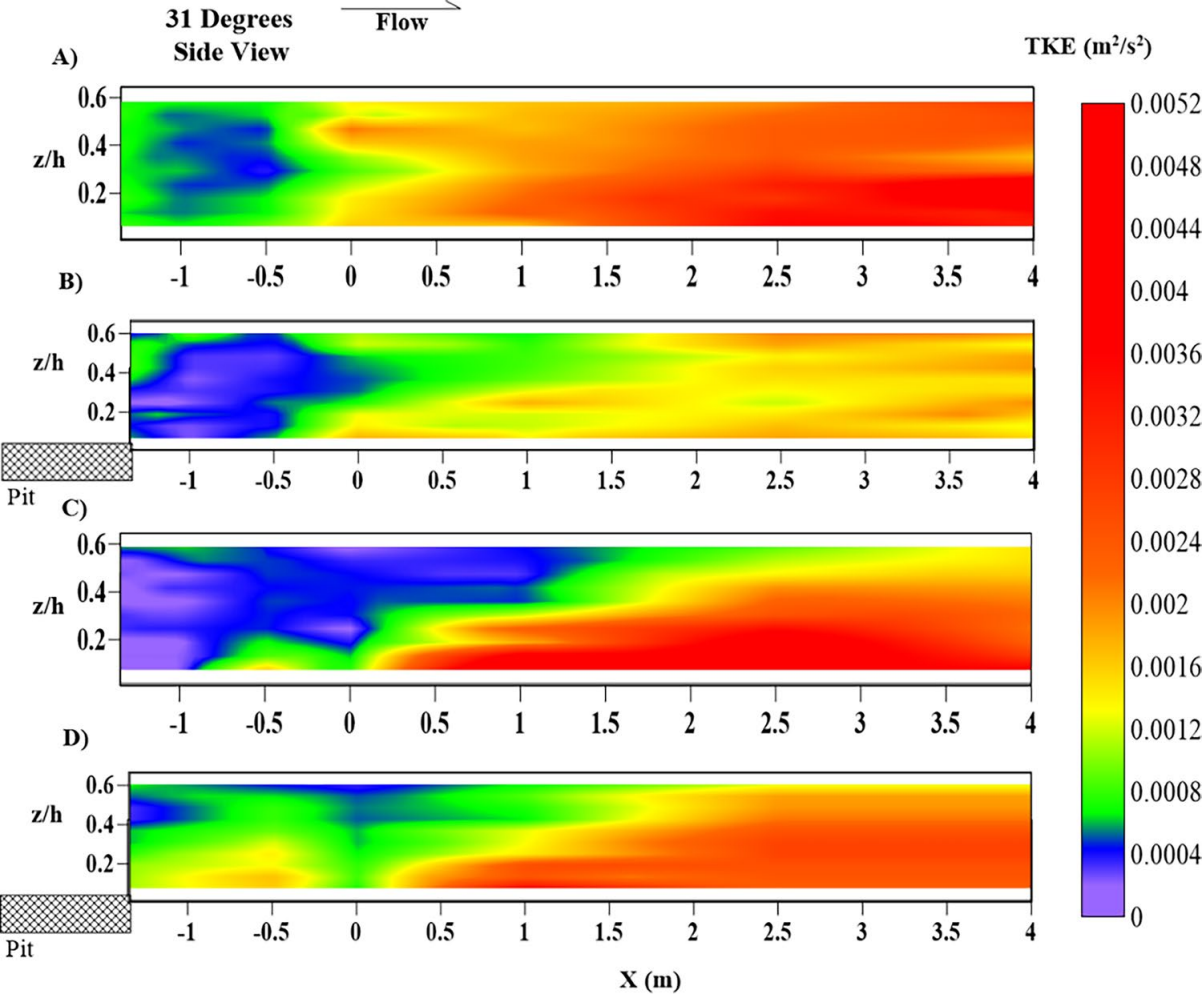
Contour of turbulent kinetic energy (TKE) k (m^2^/s^2^) longitudinal variations, at Y = 0.7 m, at the center line of the main channel representing (**A**) NPNV; (**B**) WPNV; (**C**) NPWRV; (**D**) WPWRV.

**Figure 12:**
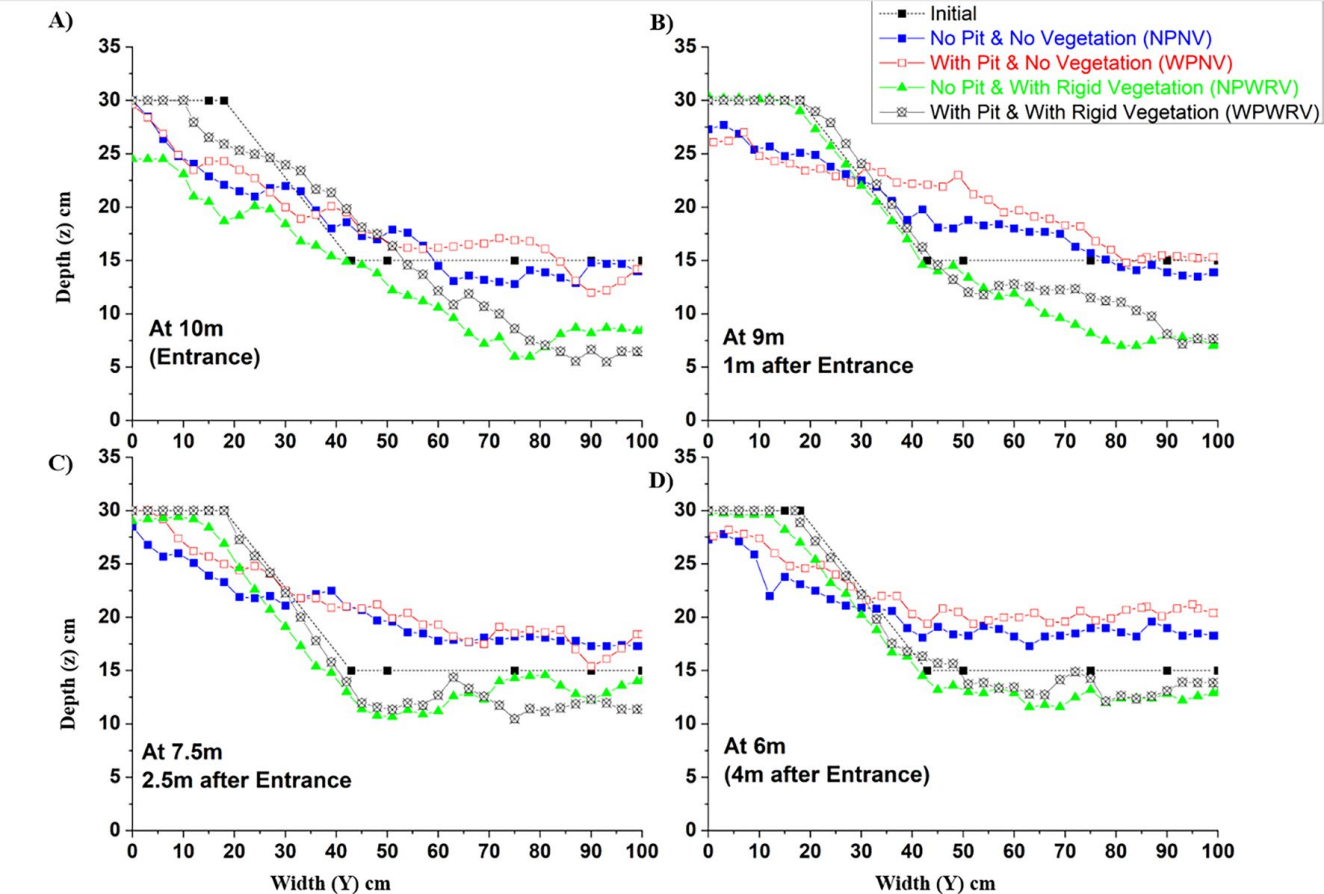
Cross-sectional profile after 24 h experimental run using point gauge for the cases of NPNV, WPNV, NPWRV, and WPWRV at (**A**) entrance; (**B**) 1 m after entrance; (**C**) 2.5 m after entrance; (**D**) 4 m after entrance into the test section along with the given initial profile of the cross-section.

**Figure 13:**
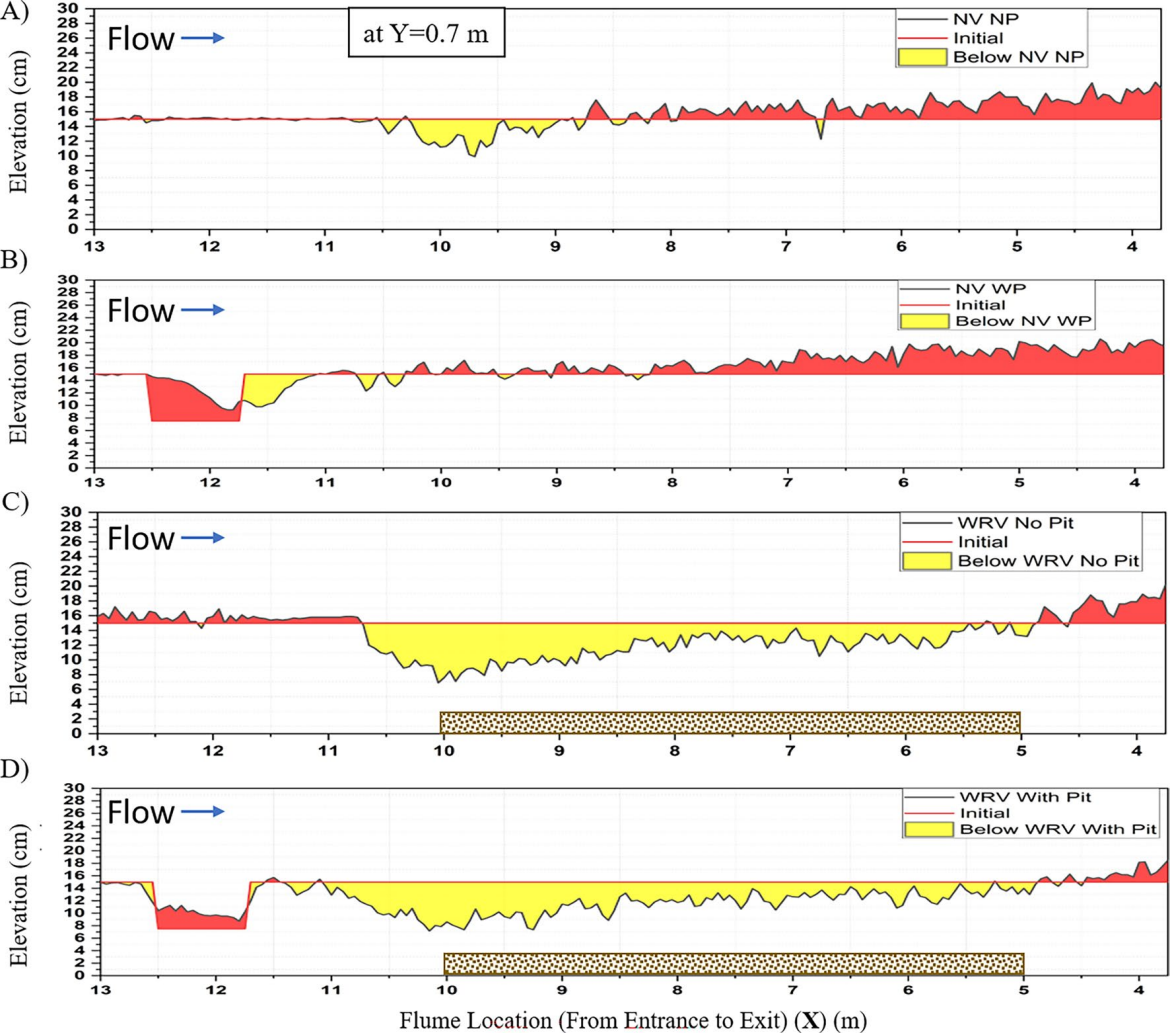
Bed elevation profile at Y = 0.7 m at the center line of the main channel of cases (**A**) NPNV; (**B**) WPNV; (**C**) NPWRV; and (**D**) WPWRV using point gauge after 24 h experimental run. The area in yellow represents the erosion, and the area in red represents the aggradation of the bed with respect to the given initial profiles. The brown dotted pattern represents the extent of rigid vegetation in the test segment.

Figure 11 shows the longitudinal variation of the TKE in the main channel at the location $Y = 0.7\;{\text{m}}$. The NPNV case suggests a higher TKE region in the main channel, which occurs in a natural channel. The shift of TKE levels from low (green) ($- 1.35\;{\text{m}} < X < 0\;{\text{m}}$) to high (red) ($X > 0\;{\text{m}}$) in Fig. 11A is due to the gradual change in the rectangular cross-section to the test section with the riverbank of 31° slope as shown in Fig. 1B, which has happened due to constriction of the flow. The WPNV case shows low TKE levels throughout as compared to the NPNV case as the high turbulence region towards the berm as the flow interacts with the berm formed at the toe of the riverbank, causing its erosion, as shown in Fig. 10C. The NPWRV case (Fig. 11C) shows an apparent increase of the TKE in the main channel as the flow diverts away from the vegetated riverbank towards the unvegetated segment. The WPWRV case in Fig. 11D shows a slight increase in turbulent kinetic energy ($- 1.35\;{\text{m}} < X < 0\;{\text{m}}$) as the flow leaves the sediment pit. The longitudinal trend of TKE suggests high turbulence in the main channel region, which shows a clear shift away from the riverbank region. This leads to the decreased erosion of the riverbank but at the cost of excessive erosion of the main channel, as seen in morphological readings presented in Figs. 12 and 13 in the following sub-section.

### Channel morphology

Figure 12 shows the cross-sections with the riverbank for each test condition after 24 h of constant flow rate of $$Q = 0.03\;{{\text{m}}^3}{\text{/s}}$$ through the test section. Four illustrations were evaluated in this investigation, each with a 31° angle (the same as the angle of repose). The point gauge readings have been plotted at four cross-sections of the test section. For all cases, the starting top width of the riverbank was 0.18 m. The entire bank's top was eroded within 24 h of the experimental run for cases NPNV and WPNV (Fig. 12). The entrance of the test section (Fig. 12A) has been affected the most due to its proximity to the upstream pit and the convergence of the flow into the test section. The pit has caused increased berm erosion, as seen in Fig. 12A,C. Additional Reynolds shear stresses are imposed on the $$ X - Y$$ sediment plane due to sand excavation, which augments downstream streamwise and transverse sediment transport.

Figure 12A,B suggests more significant bank erosion in the WPNV case due to the presence of a sand pit compared to the case NPNV^2,38^. The aggradation of the downstream segment for the cases NPNV and WPNV in Fig. 12B–D, $Y > 0.4\;{\text{m}}$, has occurred due to the sediment transported from the upstream segments by depleting the upstream bank, berm, and bed. The entire cross-section has almost leveled, which shows greater instability induced by the sediment pit to the downstream section. Figure 12C supports the results from previous research that the riverbed faces the direct effects of the sandpit, whereas the riverbank has decreased erosion. However, the bank would now face increased unsupported height for the upcoming years^2,41^. This suggests the instability in the selected test section without introducing vegetation on the riverbank.

In all profiles of Fig. 12, the vegetated sections have shown cross-sectional stability even more than the NPNV case, which is considered here as the reference when anthropogenic interference, such as sediment mining, has not occurred. The concept of inducing stability by nullifying the adverse effects of sandpit has been overcome more than expected, owing to the vegetated riverbank's flow deflection properties. However, the main channel (unvegetated), $Y > 0.5\;{\text{m}}$, has seen much higher erosion in cases of NPWRV and WPWRV due to the increased streamwise velocity, RSS, and transverse RSS, as discussed in previous sub-sections. The erosion of more than 67% of the sand bed in the main channel has been observed. The riverbank maintained its profile throughout the test segment, due to which the eroded sediments could not settle in the test segment, which was seen in the cases of NPNV and WPNV. In the case of NPWRV, the downstream section presented in Fig. 12C,D, $Y > 0.45\;{\text{m}}$, has not seen much aggradation because the sediment from the upstream bank and berm has stayed composed due to the vegetation.

Figure 13 represents the erosion (in yellow) and aggradation (in red) of the bed profile along the flow length at $Y = 0.7\;{\text{m}}$ due to the flow action of 24 h for the initial profile. The test segment lies in $10\;{\text{m}} < X < 5\;{\text{m}}$, where the brown dotted texture represents the extent of rigid vegetation on the riverbank. Figure 13A for the case NPNV shows slight erosion in the zone, which occurred when the flow entered the test section. The downstream zone $X < 9\;{\text{m}}$ experiences more aggradation than erosion due to the sediment supply from the eroded riverbank in the test segment. Similar aggradation is observed in the WPNV case, but a slight shift of the sediment pit has been observed, which agrees with the previous research^52^. Since the riverbank has maintained its profile in the NPWRV and WPWRV cases, the main disruption can be seen in the main channel. Figure 13C,D represents the center line of the main channel. The erosion from the main channel in the test segment peaks at 0.075 m at the entrance of the test section for both cases. However, the WPWRV case observed higher erosion volume than the NPWRV case in the test segment in $5\;{\text{m}} < X < 10\;{\text{m}}$. The observed erosion areas in the test segment at the center line $Y = 0.7\;{\text{m}}$ for NPWRV and WPWRV were measured to be 0.01565 m^2^ and 0.01685 m^2^, respectively. The increment of 7.66% in the erosion at the center line of the main channel shows the effects of the upstream sediment pit, which has caused an increase in shear stresses and turbulent kinetic energy of the flow, as discussed in the previous sub-sections. The data suggests that removing sediment from a river can have a significant impact on the riverbed and surrounding banks downstream. Additionally, if vegetation is introduced to combat this issue, it may further impact the main channel. This highlights the necessity for continued monitoring and regulations by stakeholders including local communities, government agencies, and environmental organizations.

## Conclusion

The flow structure of the four scenarios with nomenclature, no pit and no vegetation (NPNV); with upstream pit and no vegetation (WPNV); no pit and with rigid vegetation (NPWRV); and with upstream pit and with rigid vegetation on the riverbank (WPWRV) were examined. While the vegetation increased the streamwise velocity in the outer layer of the main channel, its combination with the upstream sediment pit influenced the near-bed streamwise velocity profile by increasing the streamwise velocity, resulting in increased sediment transport. Rigid emergent vegetation positioned equidistant in the riverbank zone reduced the streamwise velocity close to the riverbank. The rigid vegetation caused a slight increase in the head as flow in the vegetated segment tried to overcome the resistance. Increased near-bed streamwise shear stresses, brought forth by the sandpit, have accelerated sediment movement from the upstream test section to the downstream test section.

On the other hand, the vegetation did not change the near-bed shear stress but instead raised the shear stress in the outer layer. However, the combination of sandpit and riverbank vegetation caused high shear stress in the main channel, leading to its instability. Due to the sediment pit, the transverse shear stress increased in the entire cross-section, especially near the bed. It leads to the lateral transport of sediment, which assists in destabilizing the sediment particles and, eventually, reduces stability in the channel cross-section by downstream transport of the bedload with the flow. The rigid vegetation showed sharp changes from positive at the bank, negative at the interface, and positive in the main channel. The changes became even steeper with the combined effect of vegetation and sediment pit, suggesting the instability of the main channel. The upstream sediment pit caused an increase in the TKE in the near bed zone for the case WPNV as compared to the NPNV case. The location of the increased TKE was the same as that of the berm of the riverbank, leading to its erosion and eventual increased unsupported height of the riverbank. This can lead to mass failure in the upcoming season or can cause undercutting before the overhang failure. The vegetation caused an increase in TKE from the interface to the main channel. The turbulent kinetic energy for the cross-section and longitudinal section of the WPWRV case was more than that of the other three cases, which has occurred due to considerable contributions of 3D fluctuations in the flow with combined effects of vegetation and an upstream sediment pit.

The morphology of the NPNV and WPNV showed erosion of the entire bank and further aggradation in the sediment at the riverbed. This led to the approximate leveling of the whole cross-section and increased bed elevation in the downstream part of the test segment for these two cases of no vegetation. This led to the compulsion to introduce riverbank erosion prevention measures like riverbank vegetation. The NPWRV and WPWRV helped the cross-section maintain its profile throughout the test segment, but the diversion of flow away from the bank led to increased erosion of more than 67% of the riverbed in the main channel. However, the overall profile of the downstream cross-sections showed a stable riverbank with slight variation in the riverbed profile of the main channel, which necessarily means that there was not much effect on the conveyance capacity or the thalweg of the channel. The longitudinal profile of the morphological study at the center line of the main channel presented the slight forward movement of the sandpit, which aligns with the previous research. The increased aggradation downstream for the WPNV case compared to the NPNV suggests increased instability due to the sediment pit. The findings pertaining to the morphological alterations resulting from sediment dredging underscore the significance of effective regulation of dredging activities by local authorities. Such measures are essential to mitigate the potential environmental impacts of dredging and ensure sustainable development in affected areas.

The riverbank with the installed dense rigid vegetation faced minimal change, even with an upstream sandpit. The rigid vegetation contributed to the bank's increased stability but at the cost of instability of the riverbed and increased sediment transport in the main channel. Over the long term, the presence of these rigid stems will serve to protect the riverbank from erosion, while concurrently leading to an increase in sediment transport within the main channel. It is anticipated that an equilibrium state will eventually be reached, provided that there is a sustained supply of sediment from upstream. The primary contribution of this study would be to supplement the hypothesis that, as shown in the results section, rigid vegetation would have protected the riverbank but would have compromised the main channel bed profile due to the enhanced flow rate in the main channel. Conversely, sparsely planted vegetation would have been more beneficial in protecting riverbanks while maintaining the profile of the overall cross-section from the eroding effects of upstream sediment mining pit.

## Data Availability

Data will be made available on reasonable request to the corresponding author.
